# Spontaneous virus reactivation in cattle chronically infected with bovine leukemia virus

**DOI:** 10.1186/s12917-019-1908-7

**Published:** 2019-05-16

**Authors:** Juan Pablo Jaworski, Marcos Iván Petersen, Hugo Adrián Carignano, Karina Gabriela Trono

**Affiliations:** Consejo Nacional de Investigaciones Científicas y Tecnológicas (CONICET), Instituto Nacional de Tecnología Agropecuaria (INTA), Instituto de Virología. Nicolás Repetto y De los Reseros (s/n), Hurlingham (CP1686), Buenos Aires, Argentina

**Keywords:** Retrovirus, BLV, Stress, Transcriptional activation

## Abstract

**Background:**

The absence of virus expression during the chronic stage of bovine leukemia virus (BLV) infection and its reactivation upon ex vivo culture has become a long-lived Dogma. During the chronic stage of BLV infection the immune response limits viral replication and the mitotic division of latently infected cells, carrying BLV provirus, allows viral expansion and disease progression towards a lymphoproliferative disorder. Several stressor factors have been associated with animal production and handling. As natural mediator of stress, glucocorticoids are strong immunosuppressive agents; moreover, they can bind long-terminal repeat region of retroviruses and induce viral expression. In the present study, we present a case report describing the spontaneous reactivation of BLV infection in naturally infected cattle.

**Case presentation:**

In order to investigate if virus reactivation occurred in vivo during the course of BLV infection, we followed up for 328 days one Holstein cow (> 3 years) chronically infected with BLV which presented high-proviral loads. This animal was neither lactating nor pregnant. Furthermore, we investigated if a stressor stimulus, in this case the administration of a synthetic glucocorticoid (dexamethasone), could impact the course of BLV infection in three additional cattle. For the first time, we observed a high level of BLV transcripts in a total of four cattle chronically infected with BLV. The detection of viral transcripts corresponding to *pol* gene strongly suggests virus reactivation in these animals. Interestingly, this simultaneous virus reactivation was unrelated to dexamethasone treatment.

**Conclusions:**

We reported for the first time spontaneous and high level of BLV transcriptional activation in cattle chronically infected with BLV. Although virus reactivation was unrelated to dexamethasone treatment, other stressor stimuli might have influenced this outcome. Future studies will be necessary to understand these observations, since the spontaneous virus reactivation presented here might have implications on BLV pathogenesis and transmission.

**Electronic supplementary material:**

The online version of this article (10.1186/s12917-019-1908-7) contains supplementary material, which is available to authorized users.

## Background

Bovine leukemia virus (BLV) is a deltaretrovirus from the Orthoretrovirinae subfamily and Retroviridae family. BLV causes a persistent infection in cattle and in most cases this infection is asymptomatic [1]. In one third of infected animals the infection progresses to a state of persistent lymphocytosis (PL) and 1 to 10% of infected cattle develop lymphosarcoma [1]. BLV is distributed worldwide except for Western Europe, Oceania and few other countries (e.g., Israel), where BLV has been eradicated. The high prevalence of this agent is associated with a significant economic impact on dairy industry due to trade restrictions and replacement cost. Additionally, BLV negatively affects production (i.e., reduced milk production) by interfering with cattle immunity and increasing the risk of secondary infection and disease (i.e., pneumonia, diarrhoea, mastitis, etc.), as reviewed by Training et al., Bartlett et al. and Frie et al. [2–4].

During primary BLV infection, viral spread occurs via the production of virions able to infect new target cells (replicative infection stage) [5]. The expression of viral antigens induces a strong humoral and cytotoxic immune response, and such response is effective in limiting the virus replication cycle. Since anti-viral immune response persist throughout the animal’s life, during the chronic stage of infection, the integrated proviruses expand by mitosis of infected host cells carrying BLV provirus, by a process known as clonal expansion (mitotic infection stage) [5]. Supporting this hypothesis several studies support a lack of viral expression in cells with an integrated provirus and BLV transcription is almost undetectable in vivo, during the chronic stage of infection; however, the incubation of heparinized blood at 37 °C is sufficient to induce ex vivo virus reactivation in PL infected subjects [6]. Alternatively, it has been proposed that low levels of viral expression persist in a subpopulation of infected cells, and these cells are efficiently killed by the immune system [7]. In this regard, the balance between a virus attempting to proliferate and a strong immune pressure may drive the course of BLV infection in its natural host. In this scenario, the host immune response may be unable to destroy cells in which viral transcription has been completely silenced, and for that reason the viral reservoir cannot be cleared.

Here we present a case report describing the spontaneous reactivation of BLV infection in naturally infected cattle. Moreover, we performed a pilot study to investigate if a stressor stimuli, in this case the administration of a synthetic GC [dexamethasone (DEX)], had an effect inducing the reactivation of BLV chronic infection in cattle. We hypothesized that by interacting with BLV LTR and/or modulating host immunity, DEX may provoke BLV reactivation in its natural host.

### Case presentation

*Spontaneous replication of BLV in chronically infected cattle.* In order to investigate if virus reactivation occurred during the course of chronic infection with BLV we followed-up for 328 days, one single Holstein cow (> 3 years; ID# 184) which was chronically infected with BLV and presented high-proviral loads. Blood samples from this animal were obtained at days 0, 20, 52, 82, 110, 160, 165, 167, 174, 181, 188, 216, 244 and 328, and the detection of viral RNA in plasma was used to assess virus reactivation in this animal under natural conditions. Refer to the Supplementary Materials for a detailed description of all materials and methods.

**Fig. 1:**
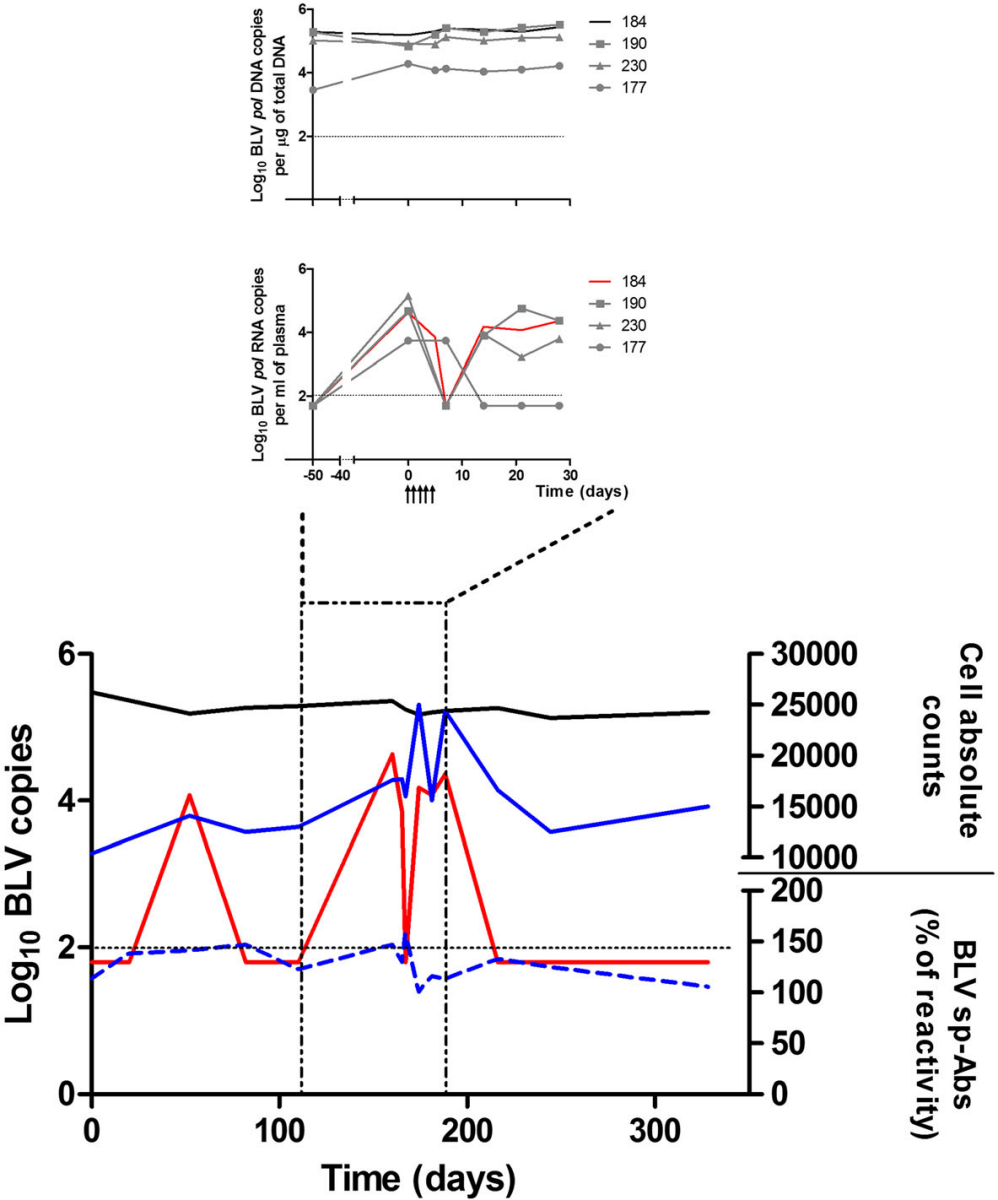
Spontaneous virus reactivation during chronic BLV infection in cattle**.** The BLV proviral load (black), BLV *pol* RNA expression (red), level of BLV-sp antibodies (blue dotted line) and the absolute counts of lymphocytes (blue solid line) were followed-up in a single Holstein cow chronically infected with BLV. BLV DNA and RNA levels are expressed as Log_10_ of copies per μg of total DNA and per ml of plasma, respectively (left axis). BLV-sp Abs were measured by ELISA and are expressed as percent of reactivity (right axis). The dotted horizontal black line represent the limit of detection of BLV qPCR (100 copies per μg). The Figure Insets correspond to a pilot study to assess the effect of a stressor *stimuli* on BLV infection in cattle. The BLV proviral load (Upper inset) and BLV *pol* RNA expression (Lower inset) were followed-up in four cattle infected with BLV; three of these cattle received DEX treatment (IDs# 177, 190 and 230) and one was used as an untreated control (Animal 184). All these parameters were measured in peripheral blood. BLV DNA and RNA levels are expressed as Log_10_ of copies per μg of total DNA and per ml of plasma. The dotted horizontal black line represent the limit of detection of BLV qPCR (100 copies per μg). Black arrows represent DEX administrations

High-proviral load in animal 184 remained stable during the whole study [Mean number of BLV copies per μg of total DNA = 182,500 (154,240 - 210,767)] (Fig. 1). Interestingly, we observed three peaks of BLV *pol* RNA expression in animal 184 (at days 52, 160 and 188), suggesting different times of virus reactivation (Fig. 1). Although virus reactivation in this animal did not have considerable impact on the proviral loads, it might have impacted the levels of total lymphocytes and BLV-specific antibodies in blood and plasma, respectively (Fig. 1).

Two years after the end of this particular study, animal 184 was euthanized. At this time, we obtained blood from this animal and a limited amount of material from lymph-node, spleen, liver, kidney and bone marrow samples (see supplementary material for details). Although this animal presented very high-proviral load for several years (> 3 years) it did not present any clinical sign. Moreover, we did not detect BLV RNA expression in plasma beyond day 188 (Fig. 1 and Table 1). Interestingly, at necropsy, animal 184 presented detectable levels of BLV RNA in spleen; in contrast, BLV proviral DNA was detected in several tissues (i.e. spleen, lymph-node, liver, kidney and bone marrow) (Table 1).

**Table 1:** Detection of BLV *pol* gene (DNA) and transcript (RNA) in tissues from animal 184

Animal ID	Tissue	BLV *pol* DNA^1^	BLV *pol* RNA^2^
184^3^	Blood	309,000	ND^4^
	Spleen	100,000	6500
	Prescapular lymph node	36,000	ND
	Liver	3500	na
	Kidney	2100	na
	Bone marrow	2000	ND

*ND* not detected

*na* not assayed

^1^BLV proviral DNA measured by qPCR (copies per μg of DNA)

^2^BLV RNA by RT-qPCR (copies per mg of tissue)

^3^DEX untreated control

^4^Plasma sample

*Dexamethasone treatment had no impact on virus expression during BLV chronic infection in cattle.* Based on our observation regarding high and spontaneous expression of BLV RNA in animal 184, we performed a pilot study to investigate if a stressor stimuli, in this case the administration of a synthetic GC (DEX), had an effect inducing the reactivation of BLV chronic infection in cattle. We hypothesized that by interacting with BLV LTR and/or modulating host immunity, DEX may provoke BLV reactivation in its natural host. For this purpose we administered 0.1 mg/kg of DEX (IV) during five consecutive days to three Holstein cattle (> 3 years of age; IDs# 177, 190 and 230) chronically infected with BLV; of note, none of these animals had detectable levels of BLV RNA in plasma before the initiation of the study (at day − 50; lower inset from Fig. 1 and Additional file 1: Table S1). Animal 184 (our Case Report animal), did not receive DEX treatment and was used as a single control. All animals were followed-up for 28 days. Unexpectedly, at day 0 (before the first administration of DEX), we detected presence of BLV *pol* RNA in the plasma from all four animals, including animal 184 (control) (Lower inset of Fig. 1). Following DEX treatment, we observed similar kinetics of BLV RNA and DNA levels in animals 184 (control), 190 and 230. Viral RNA copy numbers in these three animals reached undetectable levels by day 7 (Lower inset of Fig. 1) and by day 14 BLV RNA levels in these animals rebounded and continued this trend by day 28. Despite these fluctuations in virus RNA expression, the proviral loads in these animals remained high and stable during the 28 days of study (Upper inset of Fig. 1). The fourth animal in this group (ID: 177) showed a slightly different pattern; BLV RNA levels remained stable during the first week following DEX treatment and then reached undetectable levels by day 14 after DEX treatment (Lower inset of Fig. 1). Besides being the animal with the lowest proviral load within the group, this animal (ID: 177) also showed the lowest levels of total lymphocytes.

## Discussion and conclusions

Viral latency during BLV chronic infection in cattle and its reactivation upon ex vivo culture became a long-lived Dogma [6]. Interestingly, in the present study, we observed for the first time spontaneous in vivo BLV RNA expression in cattle chronically infected with BLV. The detection of viral transcripts corresponding to *pol* gene in cattle chronically infected with BLV suggest in vivo virus reactivation. Particularly, animal 184 presented at least three different peaks of virus reactivation in a one year time-frame. Interestingly, the fact that during the necropsy of this particular animal BLV DNA was detected in several tissues, but BLV RNA was only detected in the spleen, highlights that virus reactivation might be occurring in particular tissues without been necessarily reflected in peripheral blood or plasma.

At the beginning of the present study, our central hypothesis was that by interacting with BLV LTR and/or modulating host immunity, a stressor stimuli, in this case DEX (a synthetic GC), might provoke virus reactivation in BLV chronically infected cattle. Stress is defined as the biological response elicited when a threat to homeostasis is perceived. Several psychological and physical stressors factors are associated with animal production and handling (i.e.: intensive animal handling, transportation, heat, weaning, restraint, contact with people and exposure to novelty, fear, etc.). Animals exposed to stress stimuli present higher levels of endogenous glucocorticoids (GC) (e.g., cortisol) which in turn induce the expression and/ or repression of several genes, through binding GC-receptor (GCR) [8, 9]. In addition, it has been proposed that GC might increase viral replication of different retroviruses, including BLV [10, 11]. The effect of GCs on virus replication can be explained by a direct (i.e., interaction with the glucocorticoid response element (GRE) located in the long terminal repeat (LTR) of the virus) or an indirect effect, since GCs may modulate immune response. The role of GCs as strong immunosuppressive agents has been extensively demonstrated [12–15]. It has been shown that GC may affect host immune response by different mechanisms as is the case of the inhibition of proinflamatory citokines in macrophages [16], and a decrease in antigen presentation to CD4+ and CD8+ T cells by dendritic cells (DC) [14, 15, 17], among other mechanisms. However, the preliminary results presented here suggest that DEX had no effect in the course of BLV infection in its natural host. These results are in contrast to what had been previously described by Niermann and colleagues [11], who showed that administration of DEX augmented BLV expression in different cells lines in vitro. Although we did not observe a direct effect due to DEX administration, we cannot exclude other stressor stimuli being responsible for the virus reactivation detected in some animals during the present study. In this regard, we observed a simultaneous peak of viral RNA expression in all four animals (including a single untreated control) occurring at day 0 and before the first administration of DEX. Although this is out of the scope of the present manuscript, we must consider that other external stressor factors might have influenced this outcome. Since several stressor stimuli are usually associated with animal handling, future investigations should be focused on understanding this interesting finding. Alternatively, BLV might have evaded immune pressure through mutation of targeted epitopes and selection of scape variants, as is the case of other known retroviruses (human immunodeficiency virus type 1, equine infectious anaemia, etc.) [18, 19].

In summary, we reported for the first time spontaneous in vivo BLV transcriptional activation in cattle chronically infected with this virus. Transient virus reactivation might have implications on BLV pathogenesis (i.e.: disease progression, tumorigenesis, immunity, etc.) and transmission. Further studies are granted to study all these preliminary findings and their implications.

## Additional file

Additional file 1: **Table S1.** Detection of BLV *pol* gene (DNA) and transcript (RNA). (DOCX 29 kb)

## Abbreviations

BHV-1: Bovine herpes virus type-1
BLV: Bovine leukemia virus
DC: Dendritic cell
DEX: Dexamethasone
DNA: Deoxyribonucleic acid
ELISA: Enzyme-linked immunosorbent assay
GC: Glucocorticoid
GCR: Glucocorticoid receptor
GRE: Glucocorticoid response element
ID: Identification
IV: Intravenous
LTR: Long-terminal repeat
PL: Persistent lymphocytosis
qPCR: quantitative real-time polymerase chain reaction
RNA: Ribonucleic acid
