# Supplementary material for: Spontaneous virus reactivation in cattle chronically infected with bovine leukemia virus
Source: BMC Vet Res. 2019 May 16;15:150. doi: 10.1186/s12917-019-1908-7 (PMC6524309; doi:10.1186/s12917-019-1908-7)
Supplement: Supplementary file 1 — Table S1. Detection of BLV pol gene (DNA) and transcript (RNA). (DOCX 29 kb) [file 12917_2019_1908_MOESM1_ESM.docx]

**SUPPLEMENTAL MATERIALS AND METHODS**

***Animals and experimental design***

In the present study, we followed up one single animal (ID#184) for a period of 328 days, in order to investigate if virus reactivation occurred during the course of BLV chronic infection; the detection of viral RNA in plasma was used to assess reactivation of viral replication in this animal under natural conditions. Animal 184 was a BLV-seropositive Holstein cow (> 3 years; 600 Kg of weight) which presented high BLV proviral loads (Supplementary Table 1). The threshold between high- and low-proviral loads was set to 2,500 copies of BLV per µg of DNA, compatible with 1% cells carrying BLV provirus, as the general criteria for proviral load characterization [1]. During this particular study, animal 184 was neither in lactation nor pregnant. Blood samples from this animal were obtained at days 0, 20, 52, 82, 110, 160, 165, 167, 174, 181, 188, 216, 244 and 328. Approximately 2 years after the finalization of current study this animal was euthanized by reasons that were unrelated to our study. The necropsy of this animal was performed by the pathology-team and particular tissue samples were shared upon special request (i.e.: blood, liver, spleen, lymph-node, kidney, bone marrow) for nucleic acid extraction and detection of BLV DNA and RNA.

Additionally, we designed a pilot experiment to test the effect of a stressor stimuli, in this case DEX treatment, in BLV chronically infected cattle. For that purpose we used three additional cows chronically infected with BLV. The age and weight of these animals was 3 years (1 lactation) and 600-650 Kg, respectively. The BLV-positive status in these animals was determined by repetitive (> 1 year) assessment of BLV-specific antibodies by ELISA and the detection of BLV DNA by nPCR and/or qPCR (Supplementary Table 1). Before the initiation of this particular study (t -50 days), all animals presented high BLV proviral load. Starting at day 0, we administered 0.1 mg/kg of DEX (IV) during five consecutive days to three out of four animals with high-proviral load. This treatment has been described to be effective inducing bovine herpesvirus type 1 (BHV-1) reactivation in cattle [2]. Animal 184 (our Case Report animal), did not received DEX treatment and was used as a single control. After DEX treatment, all four animals were simultaneously followed-up for 28 days, where we looked for clinical signs, blood cell counts, BLV DNA, BLV RNA, and BLV-specific antibodies. Samples were systematically collected at day 0 (before first administration of dexamethasone), days 5 (after the last administration of dexamethasone), 7, 14, 21 and 28.

All the animals used in the present study were housed in a loose housing yard with free stalls form an experimental facility that belongs to the Centro de Investigaciones en Ciencias Veterinarias y Agronomicas (CICVyA), Instituto Nacional de Tecnología Agropecuaria (INTA) (Buenos Aires, Argentina). Animals were fed with an unmixed feeding system in which concentrates are administered separately from forages. The animals were periodically examined by the veterinarian staff. All experimental protocols, including procedures used for animal handling and sampling were approved by the Institutional Animal Care and Use Committee of the INTA-CICVyA (N#: 40/2014). The guidelines described in the Institutional Manual were followed at any time.

***Determination of BLV proviral load (BLV pol DNA)***

Peripheral blood leukocytes (PBL) were isolated from field samples by centrifugation at 1,500 g for 25 min and erythrocytes were hemolysed by osmotic shock with ultrapure H_2_O and 4.5% NaCl. After two washes in PBS, the supernatant was discarded and the cell pellet was used for extraction of genomic DNA. Tissue samples collected during the necropsy were stored in 1.5 plastic tubes. All samples were kept at -80°C until used. Total genomic DNA was extracted using High Pure PCR Template Preparation Kit (Roche, Penzberg, Germany) according to the manufacturer’s instructions. The DNA concentration was calculated using nanophotometer (Nanodrop, Thermo Fisher Sci, US). The DNA samples were stored at -80 °C until use. For the detection of cell-associated viral load, we used a SYBR Green real-time quantitative PCR (BLV qPCR) assay developed in our laboratory [3]. Briefly, each BLV SYBR qPCR reaction contained Fast Start Universal SYBR Green Master Mix (Roche), 800 nM forward and reverse primers (BLVpol5f: 5’–CCTCAATTCCCTTTAAACTA-3’and BLVpol3r: 5’–GTACCGGGAAGACTGGATTA–3’) and 50 ng DNA template. The reaction was performed on an ABI 7500 machine (Thermo Fisher Sci) with the following cycling conditions: 2 min at 50º C, 95º C for 10 min, followed by 40 cycles at 95º C for 15 s, 55º C for 15 s and at 60º C for 1 min. The plasmid pBLV1, containing BLV *pol* fragment, was used as standard; pBLV1 was kindly provided by Dr. J. Kuzmak (National Veterinary Research Institute, Pulawy, Poland). Ten-fold dilutions of this standard were made from 5x10^6^ copies µL^-1^ to 5 copies µL^-1^. Based on this dynamic range and the input of DNA, the analytical sensitivity of the assay was estimated in 100 copies per µg of total DNA. A strong and a weak positive control, as well as a negative control and a no-template control (NTC), were included in each plate. All proviral load determinations for each animal were assayed simultaneously (in the same plate) to diminish inter-plate variability.

***Determination of BLV plasma viral load (BLV pol RNA)***

Plasma and tissue viral RNA samples were extracted using High Pure RNA Isolation Kit (Roche), per the manufacturer´s instructions. The kit includes a DNAse step to remove potential contamination with genomic DNA. The RNA copy number was measured by quantitative RT-PCR (RT-qPCR). The RT reaction was performed with the High-Capacity cDNA Reverse Transcription Kit (Applied Biosystems, ThermoFisher Scientific, US), following the manufacturer´s instructions. Ten µL of extracted RNA was added to 10 µL of RT master mix, containing Multiscribe Reverse Transcriptase. To detect BLV *pol* cDNA, 2µL of RT reaction was used in the qPCR described above in a total volume of 25 µL. Each sample was run in triplicate, including 1 no RT replicate as a DNA contamination control. A positive control and a negative control, as well as two no-template control (RT-NTC and qPCR-NTC), were included in each plate. Plasma viral load analyses were performed twice, starting from the isolation of viral RNA from different plasma aliquots. All determinations of BLV RNA level for every single animal (individual plasma viral load series) were assayed simultaneously (in the same plate) as an attempt to diminish inter-plate variability.

***Detection of BLV DNA by nested PCR***

The BLV nPCR was adapted from Wu el at [4]. Briefly, the first round of the reaction contained 5x Colorless GoTaq Buffer (Promega, WI, US), 0.5 U Taq Polimerase (Promega), 5mM of each dNTP (Promega), 350 nM of each primer (Outer_Forward :5´-CAGACACCAGGGGAGCCATA-3´ and Outer_Reverse :5´-CTGCTAGCAACCAATTTCGGA-3´) and 100-1000 ng of DNA template, in a total reaction volume of 25 µL. The first reaction was performed with the following cycling conditions: 5 min at 95º C, followed by 20 cycles at 95º C for 1 min, 61º C for 1 min and at 72º C for 1 min, following a last extension of 72° C for 5 min. The second round of the reaction contained 5x Green GoTaq Buffer (Promega), 0.5 U Taq Polimerase (Promega), 5mM dNTPs (Promega), 350 nM of each primer (Inner_Forward: 5´-AGCCATACGTTATCTCTCCA-3´ and Inner_Reverse: 5´-CAGGTTAGCGTAGGGTCATG-3´) and 1µL of the product from the first round, in a total volume of 25 µL. The conditions for this second round were: 5 min at 95º C, followed by 35 cycles at 95º C for 45 s, 65º C for 45 s and at 72º C for 1 min, following a last extension of 72° C for 5 min. The presence of BLV DNA was evidenced by visualization of a 279-bp fragment corresponding to *tax* region of BLV genome. Positive and negative internal controls, as well as non-template controls (for each round of reaction) were included in each run of the assay.

***Detection of BLV-antibodies***

Plasma specific antibodies against the whole BLV viral particle were measured by indirect ELISA as described previously [5]. Briefly, ELISA plates were coated with antigen purified, from fetal lamb kidney cells persistently infected with BLV (FLK-BLV), by centrifugation on a sucrose cushion. The samples to be tested were added to the plate in duplicates. Based on preliminary data plasma samples were pre-diluted 1:40. After incubation and washing, anti-bovine IgG peroxidase conjugated was added to each well. The presence of secondary antibody was revealed with 3´, 3´, 5´, 5´;-tetramethylbenzidine (TMB) and H_2_O_2_. Reaction was stopped using 1N H_2_SO_4_ and the absorbance was read at 450 nm. Normalized results were obtained as a sample to positive ratio. A weak positive control serum (WPC) was used to calculate the ratio. The difference between the raw OD obtained for the WPC and a negative control (NC) was set to 100 %. All tested samples were referred to it and results were expressed as % of reactivity. Positivity was defined using a cut-off level of 25 %.

**REFERENCES**

1. Hopkins SG, DiGiacomo RF. Natural transmission of bovine leukemia virus in dairy and beef cattle. Vet Clin North Am Food Anim Pract. 1997;13:107–28. http://www.ncbi.nlm.nih.gov/pubmed/9071749. Accessed 13 Sep 2018.

2. Lemaire M, Schynts F, Meyer G, Georgin JP, Baranowski E, Gabriel A, et al. Latency and reactivation of a glycoprotein E negative bovine herpesvirus type 1 vaccine: influence of virus load and effect of specific maternal antibodies. Vaccine. 2001;19:4795–804. http://www.ncbi.nlm.nih.gov/pubmed/11535332. Accessed 14 Sep 2018.

3. Petersen MI, Alvarez I, Trono KG, Jaworski JP. Quantification of bovine leukemia virus proviral DNA using a low-cost real-time polymerase chain reaction. J Dairy Sci. 2018. doi:10.3168/jds.2017-14253.

4. Wu D, Murakami K, Morooka A, Jin H, Inoshima Y, Sentsui H. In vivo transcription of bovine leukemia virus and bovine immunodeficiency-like virus. Virus Res. 2003;97:81–7. http://www.ncbi.nlm.nih.gov/pubmed/14602199. Accessed 13 Sep 2018.

5. Trono KG, Perez-Filgueira DM, Duffy S, Borca M V, Carrillo C. Seroprevalence of bovine leukemia virus in dairy cattle in Argentina: comparison of sensitivity and specificity of different detection methods. Vet Microbiol. 2001;83:235–48. http://www.ncbi.nlm.nih.gov/pubmed/11574172.

**SUPPLEMENTAL DATA**

**Supplemental Table 1. Detection of BLV *pol* gene (DNA) and transcript (RNA)**

| Animal ID | Breed | Age | Type of infection | ELISA BLV ^1^ | nPCR  BLV | -50 days | |  | 0 day | |  | 5 days | |  | 7 days | |  | 14 days | |  | 21 days | |  | 28 days | | |
| --- | --- | --- | --- | --- | --- | --- | --- | --- | --- | --- | --- | --- | --- | --- | --- | --- | --- | --- | --- | --- | --- | --- | --- | --- | --- | --- |
|  |  |  |  |  |  | DNA^2^ | RNA^3^ |  | DNA | RNA |  | DNA | RNA |  | DNA | RNA |  | DNA | RNA |  | DNA | RNA |  | DNA | RNA | |
| 184^4^ | Holstein | >3 years | natural | + | + | 194,120 | ND |  | 153,352 | 42,840 |  | 203,130 | 7,140 |  | 248,515 | ND |  | 230,577 | 15,120 |  | 196,383 | 11,760 |  | 276,633 | 23,100 | |
| 190 | Holstein | >3 years | natural | + | + | 191,650 | ND |  | 68,064 | 47,460 |  | 158,382 | na |  | 258,768 | ND |  | 193,805 | 7,980 |  | 265,088 | 57,540 |  | 329,144 | 23,520 | |
| 177 | Holstein | >3 years | natural | + | + | 2,830 | ND |  | 19,146 | 5,460 |  | 11,964 | na |  | 13,373 | 5,460 |  | 10,724 | ND |  | 12,485 | ND |  | 16,305 | ND | |
| 230 | Holstein | >3 years | experimental | + | na | 103,760 | ND |  | 83,003 | 142,800 |  | 78,924 | na |  | 133,550 | ND |  | 103,140 | 8,820 |  | 127,085 | 1,680 |  | 132,859 | 6,300 | |
|  |  |  |  |  |  |  | | | | | | | | | | | | | | | | | | | |  |
| ND: not detected  na: not assayed  ^1^ more than two positive ELISA; starting 1 year before the initiation of the current study  ^2^ BLV proviral DNA measured by qPCR (copies per µg of DNA)  ^3^ BLV RNA by RT-qPCR (copies per ml of plasma)  ^4^ DEX untreated control | | | | | | | | | | | | | | | | | | | | | | | | | |  |
